# Acidic Osteoid Templates the Plywood Structure of Bone Tissue

**DOI:** 10.1002/advs.202304454

**Published:** 2023-12-19

**Authors:** Marc Robin, Chakib Djediat, Arnaud Bardouil, Niki Baccile, Camille Chareyron, Ivo Zizak, Peter Fratzl, Mohamed Selmane, Bernard Haye, Isabelle Genois, Jean‐Marc Krafft, Guylène Costentin, Thierry Azaïs, Franck Artzner, Marie‐Madeleine Giraud‐Guille, Paul Zaslansky, Nadine Nassif

**Affiliations:** ^1^ CNRS, Sorbonne Université, Collège de France Laboratoire Chimie de la Matière Condensée de Paris (LCMCP) Paris F‐75005 France; ^2^ Muséum National d'Histoire Naturelle UMR CNRS 7245, Bâtiment 39, CP 39, 57 rue Cuvier Paris 75231 France; ^3^ Université de Rennes, CNRS Institut de Physique de Rennes (IPR) Rennes F‐35000 France; ^4^ Helmholtz‐Zentrum Berlin für Materialien und Energie – Speicherring BESSY II Albert‐Einstein Str. 15 D‐12349 Berlin Germany; ^5^ Department of Biomaterials Max Planck Institute of Colloids and Interfaces am Mühlenberg 1 14476 Potsdam Germany; ^6^ Institut des Matériaux de Paris Centre Sorbonne Université Paris F‐75005 France; ^7^ Sorbonne Université, CNRS Laboratoire Réactivité de Surface (LRS) Paris F‐75005 France; ^8^ Department for Operative Preventive and Pediatric Dentistry Charité – Universitätsmedizin Berlin Aßmannshauser Str. 4–6 14197 Berlin Germany

**Keywords:** acidity, biomineralization, bone, collagen, liquid‐crystal, osteoid, plywood

## Abstract

Bone is created by osteoblasts that secrete osteoid after which an ordered texture emerges, followed by mineralization. Plywood geometries are a hallmark of many trabecular and cortical bones, yet the origin of this texturing in vivo has never been shown. Nevertheless, extensive in vitro work revealed how plywood textures of fibrils can emerge from acidic molecular cholesteric collagen mesophases. This study demonstrates in sheep, which is the preferred model for skeletal orthopaedic research, that the deeper non‐fibrillar osteoid is organized in a liquid‐crystal cholesteric geometry. This basophilic domain, rich in acidic glycosaminoglycans, exhibits low pH which presumably fosters mesoscale collagen molecule ordering in vivo. The results suggest that the collagen fibril motif of twisted plywood matures slowly through self‐assembly thermodynamically driven processes as proposed by the Bouligand theory of biological analogues of liquid crystals. Understanding the steps of collagen patterning in osteoid‐maturation processes may shed new light on bone pathologies that emerge from collagen physico‐chemical maturation imbalances.

## Graphical abstract

Extensive analyses of sheep bone biopsies ascertain that the deeper non‐fibrillar osteoid is organized in an acidic liquid‐crystal cholesteric geometry. Such acidic domains, most likely stabilized by glycosaminoglycans and citrate, foster collagen assembly from molecular into fibrillar plywood textures, a hallmark of many bone extracellular matrices. The results show that beyond cellular control, physicochemical thermodynamic processes drive bone maturation.

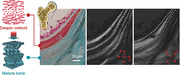

## 1 Introduction

A hallmark of many bones is the presence of domains containing mineralized collagen fibrils arranged in a twisted plywood architecture. This well‐known pattern is fairly common in different extracellular matrices (ECMs) and has emerged as one evolutionary strategy to produce resilient structural materials in nature.^[^ ^1^ ^]^ Bone is unique in that this architecture is both mineralized and dynamically created: either *de novo* (during modeling) or when tissue is replaced (by remodeling). In both cases, mineralization occurs only after the establishment of a fibrillar organic ECM known as osteoid.^[^ ^2^ ^]^ This soft tissue, produced by a layer of osteoblasts, comprises mainly collagen though it also contains non‐collagenous proteins (NCPs)^[^ ^3^, ^4^, ^5^ ^]^ and glycosaminoglycans.^[^ ^6^, ^7^ ^]^ The biological processes involved in osteoid creation have been extensively described highlighting the important role that osteoblasts have in setting up the extracellular compartment,^[^ ^8^, ^9^ ^]^ with descriptions of two distinct layers observed in many animals and in certain sample preparations.^[^ ^7^ ^]^ Osteoid has often been described as a disorganized soft material^[^ ^6^ ^]^ that gradually densifies^[^ ^10^, ^11^ ^]^ and transforms into highly ordered mature bone (MB).^[^ ^12^ ^]^ But just how the twisted plywood pattern of collagen fibrils^[^ ^13^ ^]^ emerges in osteoid in vivo has actually never been shown.

Based on in vitro models and tissue microscopy, different texturing mechanisms of twist have been proposed. These include i) the continuous rotation of osteoblasts within the plane that they establish, with synergistic collagen production,^[^ ^14^ ^]^ ii) the transfer of cellular tension into the ECM with pre‐stressed collagen fibrils appearing during tissue growth,^[^ ^15^, ^16^ ^]^ or iii) compaction and sliding of pre‐aligned procollagen molecules released from micro‐sized intracellular vesicles.^[^ ^17^ ^]^ Though these proposals are very different one from another, they share the premise that the fibrillar layout in MB is created by or through forces generated by the formative osteoblast cells. Importantly, whereas observations from the literature might explain 2D lateral fibrillar packing,^[^ ^18^ ^]^ none of the existing theories provides experimental evidence for the appearance of the long‐range and 3D twisted fibril arrangements,^[^ ^19^, ^20^, ^21^, ^22^, ^23^ ^]^ with a geometry often compared to cholesteric (helicoidal) liquid crystals (LC).^[^ ^24^ ^]^ An alternative yet complementary possibility is that a LC state of matter is created in bone.^[^ ^16^, ^19^, ^25^ ^]^

Here, we show that the deeper osteoid is organized in a cholesteric liquid‐crystal geometry. We identify that this domain previously described as basophilic and non‐fibrillar is in fact made of collagen in its molecular form enriched with acidic glycosaminoglycans. This indicates that this chemical environment creates an extracellular compartment in which collagen fibril‐twist can mature. We hypothesize that this deeper osteoid layer is a LC phase essential for establishing the plywood structure found in many bone tissues. We combine histology and polarized light microscopy (PLM), X‐ray microbeam small‐angle X‐ray scattering (SAXS), and X‐ray fluorescence (XRF) mapping with second harmonic generation (SHG) confocal laser‐scanning and electron microscopy, both scanning (SEM) and transmission (TEM). Based on our observations of freshly‐extracted bone biopsies from skeletally‐mature sheep,^[^ ^26^ ^]^ and by comparison with self‐assembly collagen/apatite in vitro essays,^[^ ^27^ ^]^ we propose that low pH is a potent modulator of fibrillogenesis in the extracellular matrix directly contributing to plywood patterning. Our findings provide missing direct evidence for the crucial role of the cholesteric phase during texturing in bone.^[^ ^24^, ^28^ ^]^

## 2 Results and Discussion

### 2.1 Texture and Composition of the Non‐Fibrillar Deeper Osteoid

**Figure 1:**
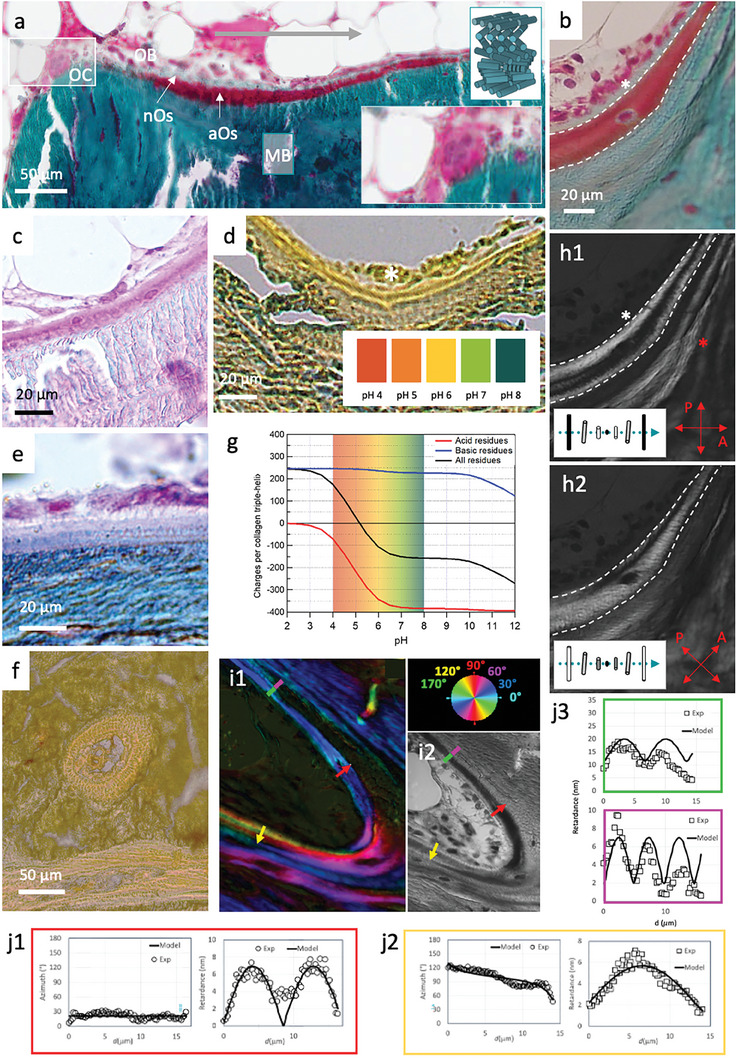
Acidic osteoid texture and composition by optical microscopies. Light microscopy overview a) of bone histological sections stained with Goldner trichrome. Acidic osteoid tissue (aOs), osteoclasts (OC), and osteoblasts (OB) nuclei are stained in deep red. Collagen fibrils in mature bone (MB) and in neutral osteoid (nOs) are stained in blue/green. A schematic representation of the mineralized collagen fibrils arrangement (in a twisted plywood model) is shown in the upper inset. The white marked rectangle is enlarged in the lower inset. b) Similar staining of a magnified bone region showing aOs (between the white dashed lines) and nOs (white ^*^). Additional bone components are visible in bone histological sections colored with c), basic fuchsin staining, d), a universal pH indicator essay observed by DIC which reveals the presence of glycosaminoglycans and a lower pH in aOs compared to the surrounding nOs and MB tissues, and e), Alcian blue/Ziehl fuchsin double‐stain reveals acidic glycosaminoglycans (violet) in the aOs. f), Histological thin section of compact bone colored with a universal pH indicator essay (color chart in d), observed by DIC. g), calculated charge per collagen triple‐helix plotted as a function of pH. Collagen molecules are slightly negative in the range of pH 5–6. h) polarization images of (b). Comparison between light (b) and polarized light h1,h2) observations demonstrates the absence of birefringence in the nOs and reveals the presence of thin alternating bright and dark bands in the aOs and in the immediately adjacent MB (red ^*^). The schematic represents the resulting intensity of transmitted light when the optical axis lies between the crossed polarizers at 0° (h1) and 45° (h2). The blue dotted line denotes the helical axis. i) Histological section showing twisted plywood motifs in MB and aOs by quantitative LC‐polscope, and observed i2), by light microscope. j1,j2) Retardance and Azimuth of the birefringence quantified on the red and yellow arrows, respectively thanks to the axis orientation (color wheel). A cholesteric model (see text and Experimental Section) well fits the experimental measurements. j3) Retardance of the birefringence quantified on the green (aOs) and violet (MB) lines which are continuous.

By conventional histology in sheep bone biopsies, the preferred orthopedic clinical model,^[^ ^29^ ^]^ we demonstrate double layering of the osteoid. Goldner trichrome stained sections (trabecular **Figure** **1**a,b; Figure S1, Supporting Information) identify the cytoplasm in red and the collagen fibrils in green–blue shades. The sequence of well‐described biological processes is reproduced in the histological slide shown in Figure 1a where the biological events are seen from left to right (arrow) revealing spatially and temporally distinct tissue‐formation stages that are typical for bone remodeling. Osteoclasts (OC) can be identified due to the multinucleate features and large size, (ca. 100 µm) and following their activation (lower inset Figure 1a; Figure S2, Supporting Information), osteoblasts (OB) appear. These later aggregate along the bone surface forming 2D sheets where they secrete a pH‐neutral osteoid (nOs). Basic fuchsin staining (magenta, Figure 1c; Figure S3a, Supporting Information) is indicative of acidic moieties which are further complemented by impregnation with a universal pH indicator essay (Figure 1d; Figure S3b, Supporting Information). Put together they highlight the co‐existence of a distinct acidic region in the osteoid in close proximity to the nOs. This double layer configuration was previously identified and described as a deeper basophilic and non‐fibrillar osteoid layer.^[^ ^6^, ^7^ ^]^ The pH indicator stain essay identifies a pH of ≈5–6, in domains that correspond to this deep osteoid (aOs). Alcian blue/Ziehl fuchsin double‐stain in an adjacent slice shown in Figure 1e, further demonstrates one possible source of this acidity, the presence of acidic glycosaminoglycans stained in violet in the aOs. These and other molecules (e.g., citrate^[^ ^30^, ^31^ ^]^ known to be bound to apatite mineral^[^ ^32^ ^]^ and to collagen^[^ ^33^ ^]^) are likely to establish an extracellular acidic domain. Low pH in fact is not rare in bone tissue, for example during the resorption process by osteoclasts (Figure 1a; Figure S2, Supporting Information) (*i.e*., ≤ 4).^[^ ^34^ ^]^ We further note that thick acidic domains can also be observed during healing (e.g., around bone defects as shown for example in Figure S4, Supporting Information). Similar domains are also identifiable in compact bone (see Figure 1f; Figure S5, Supporting Information) where the pH stain reveals acidity, identified in yellow, in the otherwise greenish, higher pH mature bone.

The establishment of stabilized proton‐rich domains in the deeper osteoid may be required to slow down fibrillogenesis through competition of electrostatic interactions at low pH.^[^ ^35^ ^]^ Indeed, negative charges of collagen due to acidic residues (red, Figure 1g) are known to decrease by a ratio of 2 from pH 7 to 5 while positive charges of the basic residues remain constant (blue, Figure 1g). In this manner, electrostatic interactions between basic‐acid residues diminish with decreasing pH.

The molecular arrangement of the aOs domain was analyzed by means of PLM (Figure 1 h = Figure b seen by PLM at 0° (1h1) and after rotation of the polars (1h2)). This method reveals alternating birefringent bright and dark bands (red ^*^ in Figure 1h) that are typical for bone tissue and are characteristic of the twisted plywood organization of collagen fibrils^[^ ^13^, ^36^, ^37^ ^]^ (schematically illustrated in the upper inset in Figure 1a). The nOs is not birefringent (identified by the white star, compared panels 1 and 2) whereas a birefringent helicoidal plywood pattern is always observed in aOs domains (identified between the white dashed lines). This is surprising because the deeper layer has been described as being non‐fibrillar.^[^ ^6^ ^]^ We also observe continuous extinction of polarized light during rotation of the polars between aOs and MB which provides evidence that there is a continuity in the molecular ordering between these two domains. Importantly, alternating fringes are also observed with cholesteric LC phases made of acid‐soluble collagen molecules in vitro.^[^ ^36^, ^38^ ^]^

We used an LC‐polscope^[^ ^39^, ^40^ ^]^ to quantify the retardance and collagen orientation (color wheel in Figure 1i) and to show the structural continuity of the birefringence patterns observed in the fully‐mineralized MB and the neighbouring aOs (Figure 1i1,2). The results are confirmed by simulations of birefringence, where profiles of cholesteric phases are determined from the pitch vector intersecting the observation cutting plane at an angle *θ*°. The twisted plywood geometrical model makes it possible to determine both molecular orientation and pitch (see Experimental Section). Three regions in the samples are used to demonstrate how ordering in the aOs translates into patterns of retardance (Figure 1i1,j1,2, and green panel in Figure 1j3). Changes in orientations of the cholesteric pitch perfectly correspond to the simulations (Figure S6, Supporting Information). When the cholesteric pitch vector is in the section plane (red line, *θ*° = 0), the azimuth is constant and the retardance exhibits a sin^2^ shape (Figure 1j1). The fitted half‐pitch is not constant, as it varies with different cutting orientations. In the example aOs region shown (red arrow), the half pitch is ≈8.5 µm wide. This thickness of lamellae fits very well with previous reports of half pitch of twisted plywood structures.^[^ ^41^, ^42^ ^]^ When the cutting plane is off‐axis (for example the yellow arrow), the pitch vector is out of plane. The retardance and azimuth yield more complex curves (Figure 1j2) that match a tilt *θ*° = 11.5°, and a larger half‐pitch of ≈17 µm. Note how, in the transition into mature bone (green–purple line), the fitted half‐pitch slightly decreases from the aOs (≈6.8 µm, green panel in Figure 1j3) to MB (≈5 µm, violet panel in Figure 1j3).

All of the above results perfectly match previous observations from the literature and suggest that acidic moieties stabilize a more acidic pH in the deep osteoid. It is known that collagen fibrils dissolve in acidic solutions in vitro.^[^ ^38^ ^]^ It is also known that the cleavage of C and N terminals of procollagen triple‐helices secreted by the cells triggers fibrillogenesis that requires neutral pH,^[^ ^43^ ^]^ as is found in the nOs. Our findings provide good indications for the existence of an LC state of matter in the aOs. Consequently, we propose that acidification of the matrix slows down fibrillogenesis and induces formation of cholesteric domains that are crucial for the emergence of the twisted plywood organization.

### 2.2 Collagen Orientation and Ultrastructure

**Figure 2:**
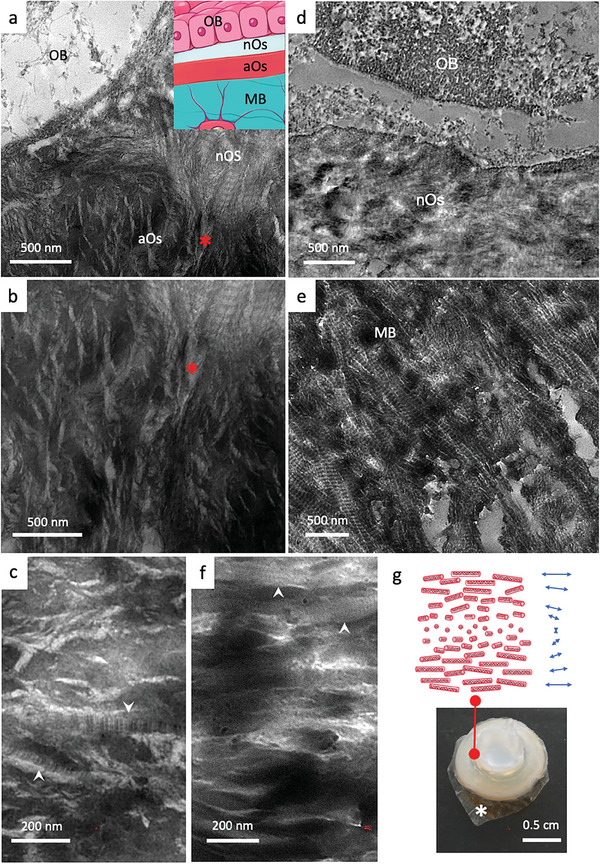
Investigations by TEM of collagen ultrastructure in bone tissues (osteoid, mature bone) and in synthetic acidic collagen mesophases which form physical gels. a,b) TEM micrographs of osteoid domains (nOs and aOs) in close proximity with an osteoblast (OB) (the red ^*^ identified the same feature in adjacent regions) show that collagen fibrils are typical for the nOs but hardly found in the aOs. The inset in a is a schematic representation of a histological bone section colored with Goldner's trichrome stain. From top to down, the monolayer that forms osteoblasts (OB), the neutral osteoid (nOs), the acidic osteoid tissue (aOs), and mature bone (MB). c) aOs appears denser to electrons with parallel packing of non‐fibrillar structures. Some fibrils are rarely observed (white arrows). Micrograph in d) shows the newly secreted nOs with randomly oriented cross‐striated fibrils in contact with an OB. In e) the classical anisotropic packing of mineralized cross‐striated fibrils in MB is observed, i.e., arced pattern revealed by an oblique section performed on the twisted plywood organization. f) in vitro lyotropic acidic solution of collagen molecules concentrated in the presence of apatite ion precursors exhibit textural similarities with aOs domains (c) are observed with locally aligned domains of collagen molecules and occasional fibrils (white arrows in c and f). g) A schematic representation of the spatial organization of collagen molecules shows a long‐range helical organization of collagen triple helices (cholesteric phase). Image of a highly concentrated acidic solution of collagen molecules (≈250 mg mL^−1^) containing apatite ion precursors (calcium, phosphate, and carbonate ions). Collagen solutions are no longer fluid at such concentration owing to their high viscosity. The sample is supported by a dialysis membrane (^*^).

The deeper osteoid has already been demonstrated to exist in many bones and it has been described as a non‐fibrillar materials.^[^ ^6^ ^]^ To further confirm its nature and to investigate its ultrastructure, TEM was performed using uranyl‐stained ultrathin sections (**Figure** **2**a–f; Figure S7, Supporting Information). The aOs appears dense with a layered smooth architecture (Figure 2a,b; FigureS7a, Supporting Information) and indeed contains only sparse large fibrils (arrows in Figure 2c; Figure S7a, Supporting Information), which is different from the typical fibrillar appearance of the nOs (Figure 2a,d) and MB (Figure 2e). Interestingly, the aOs texture resembles lyotropic (cholesteric) acidic solutions of collagen studied in vitro^[^ ^38^ ^]^ (Figure 2f,g) with aligned non‐fibrillar domains and only a few fibrils observed locally (Figure 2f; Figure S7b, Supporting Information, fibrils identified by arrows). The lack of presence of other organic constituents (e.g., glycosaminoglycans) in the synthetic sample may be a reason for the different appearance of that collagen. Strikingly, it has been previously shown that collagen molecules can precipitate into fibrils spontaneously even at low pH at concentrations exceeding 150 mg mL^−1^.^[^ ^44^ ^]^ Since collagen solutions are extremely viscous at high concentrations at which they form a physical gel (Figure 2g), our finding strongly suggests that the aOs is a cholesteric collagen‐based gel.

**Figure 3:**
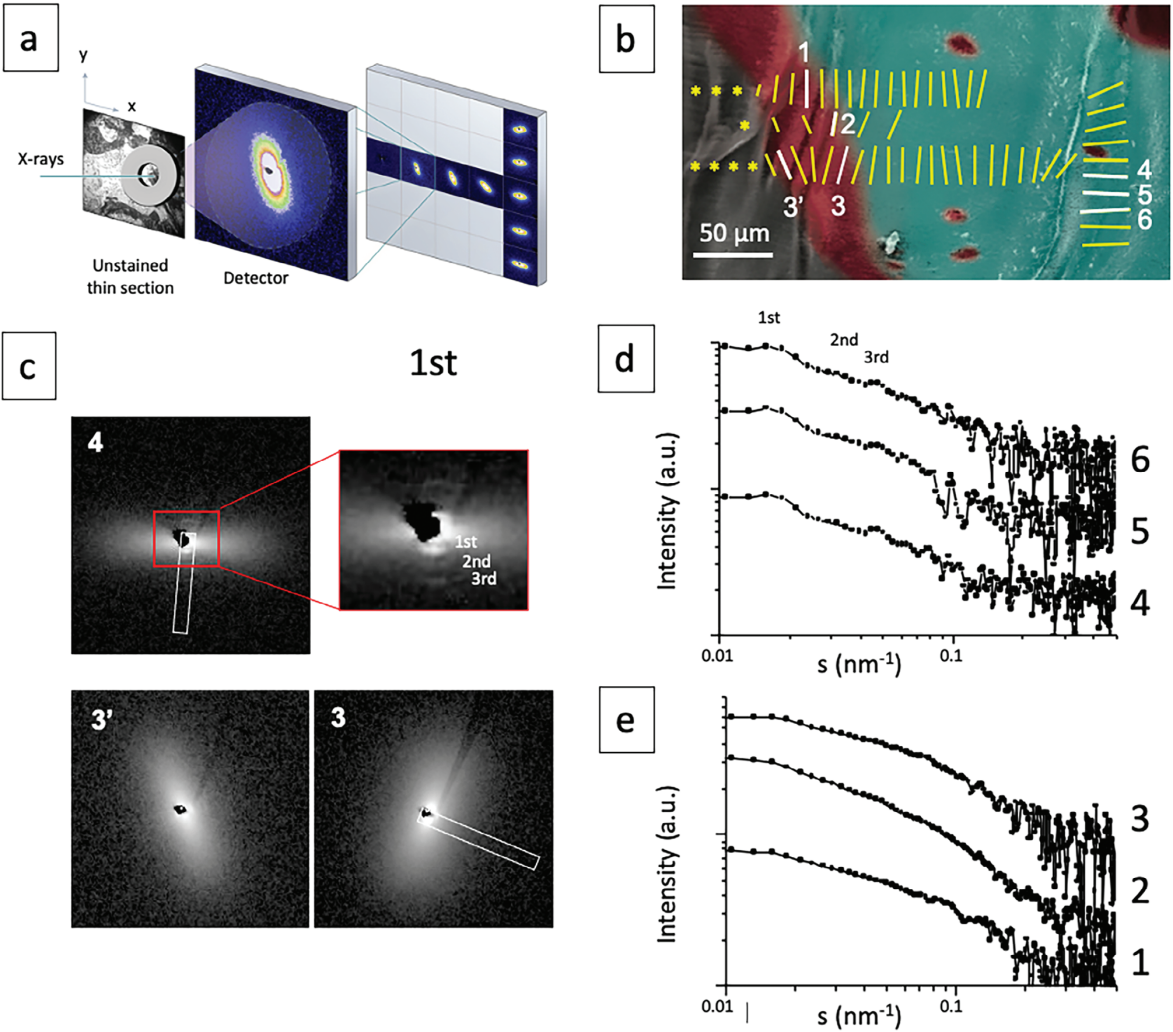
Collagen orientation by SAXS. a) The aOs and surrounding tissues is identified optically with a metal ring on the unstained serial histological thin section. The sample is scanned with a microfocus beam along a vertical and horizontal axis (x,y arrows) and a diffraction pattern is recorded at each point. b) Scattering patterns are schematically represented on the corresponding colored SEM image (blue and red colors represent MB and aOs, respectively). The signal orientation (direction of the bars) and degree of alignment (length of the bars) are shown as well as the locations of the SAXS line scans (a 90° rotation of the yellow bars corresponds to the direction of the fibrils, see Figure S8); a continuity in signal orientation is observed between mature bone (MB) and acidic osteoid (aOs). In contrast, there is no SAXS signal (yellow stars) beyond the acidic osteoid. c) 2D SAXS pattern 4 and 3‐3′ corresponds to a signal recorded for MB and aOs in (b) (white bars), respectively. The axial orientation is highlighted by a white rectangle and the red rectangle in 4 corresponds to the enlarged section of the central scattering contrast. d,e) Series of SAXS patterns extracted from the MB (d) and aOs (e) domains from (b). The profiles 4–6 are typical for fully mineralized bone domains where only the 1st‐3rd orders of collagen are observed, in contrast to profiles 1–3, extracted from the aOs domain.

To identify the orientation of collagen across the molecular aOs and fibrillar MB domains, micron‐sized synchrotron X‐ray beams were used to collect SAXS on the thin biopsy sections (**Figure** **3**a–e). Sites of X‐ray measurement (Figure 3a) were identified on histologically stained adjacent sections observed under light microscope (Figure S8a,b, Supporting Information). To avoid possible artifacts of the histological staining,^[^ ^45^ ^]^ SAXS was performed only on unstained slices (identified by metal ring in Figure 3a; Figure S8c,d, Supporting Information). The resulting SAXS measurements (Figure S8e, Supporting Information) reveal distinct zones in the osteoid. In the overlay Figure 3b, the blue and red domains identify sites of MB and aOs, respectively. Anisotropic scattering was observed in measurement spots all across the non‐mineralized and mineralized bone regions, with well‐developed SAXS signal and gradual (vs abrupt) changes in the scattering orientation (yellow lines, e.g. compare panels 3 to 3′). The scattering in regions of MB (Figure 3c4; Figure S8e, Supporting Information), similar to previous reports,^[^ ^46^ ^]^ is typical of highly mineralized fibrillar domains.^[^ ^47^, ^48^, ^49^ ^]^ This is shown by the 67 nm reflection and the corresponding harmonics of collagen periodicity observed in the 2D SAXS pattern (Figure 3c4, red rectangle); the scattering contrast of the 1st and 3rd order reflections corresponds to fibrillar ordering and is enhanced by the distribution of mineral crystals along the collagen fibrils^[^ ^48^, ^49^ ^]^ (Figure 3d4, which corresponds to the 1D profile of the collagen SAXS signal inside the white rectangle, Figure 3d6). Note that repeated X‐ray scattering measurements within aOs domains (Figure 3c3, inside the white rectangle, and Figure S8e, Supporting Information) never showed scattering signals from fibrils (see example Figure 3e1–3) suggesting that the molecules have local order but that most of the collagen is not fibrillar as seen by TEM. Yet, the SAXS patterns also show a continuity in the collagen ordering across the aOs/MB interface, a finding that matches our observations by PLM and LC‐Polscope.

### 2.3 Structure‐Formation Relationships

**Figure 4:**
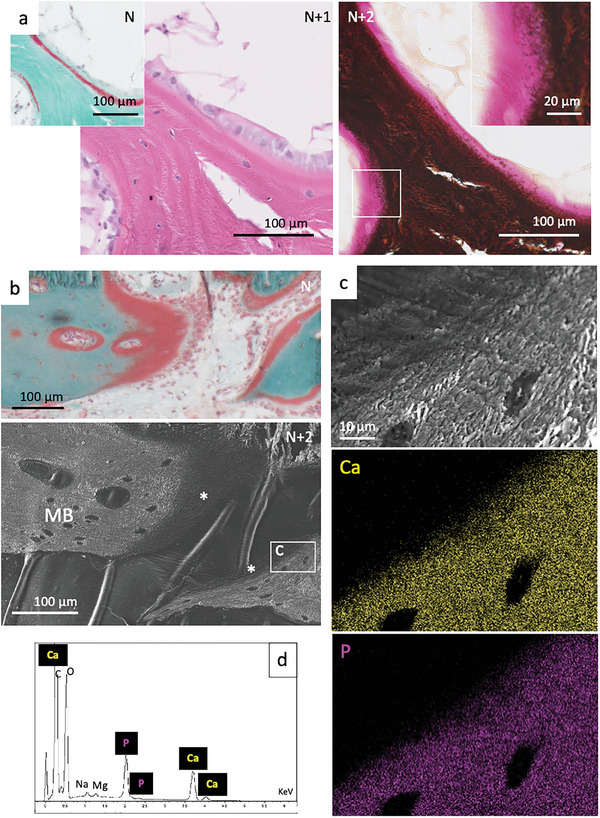
Mineralization and elemental mapping near osteoid domains. a) Observations by optical light microscopy of successive bone histological thin sections from the same region colored with Goldner trichrome (N), haematoxylin and eosin (N+1) and von Kossa (N+2) stains. The inset in (N+2) corresponds to the enlarged section of the white rectangle where a gradient in mineralization (dark deposits) is clearly identified from the non‐mineralized tissue (pink). b) Observations by optical light microscopy of a bone histological thin sections colored with Goldner's trichrome stain (N) and a subsequent section (N+2) by SEM. SEM micrograph shows a smooth texture for the acidic osteoid domain (^*^) which is distinguishable from the fibrous collagen of the surrounding bone (MB). The white rectangle indicates the enlarged section that is shown in c) and analyses by EDX d). The elemental mapping of the area shows the presence of calcium (Ca in yellow) and phosphorus (P in purple) gradients.

Complementary stains of successive histological sections from the exact same tissue regions provide insights into the aOs components (**Figure** **4**a, adjacent slices N, N+1, and N+2). The use of standard haematoxylin and eosin stains (N+1), possibly due to their pH, shows no difference in protein composition between osteoid and MB (N+2). The mineralization front is clearly identifiable by the change from pink to black, observed by von Kossa staining. Higher magnification observations (inset in N+2) show that a mineral gradient localizes at the margin of the aOs. Similar results were shown for human bone by 3D FIB‐SEM.^[^ ^50^ ^]^ Successive slices imaged by SEM and histologically by Goldner trichrome stain (Figure 3b), identify the mineralized and non‐mineralized domains of the aOs (^*^) and MB. Osteoid which comprises lower atomic number (Z) elements exhibits a darker, smoother appearance compared to mineralized MB (lower panel in Figure 4b and upper panel in Figure 4c). Analysis by energy‐dispersive X‐ray spectroscopy (EDX) and elemental mapping at higher magnification (Figure 4c,d), confirms the existence of a gradient of calcium (Ca in yellow) and phosphorus (P in purple) across the aOs/MB interface (ca. 10 µm). The gradient corresponds to a transition from the acidic aOs to the neutral‐pH MB.

**Figure 5:**
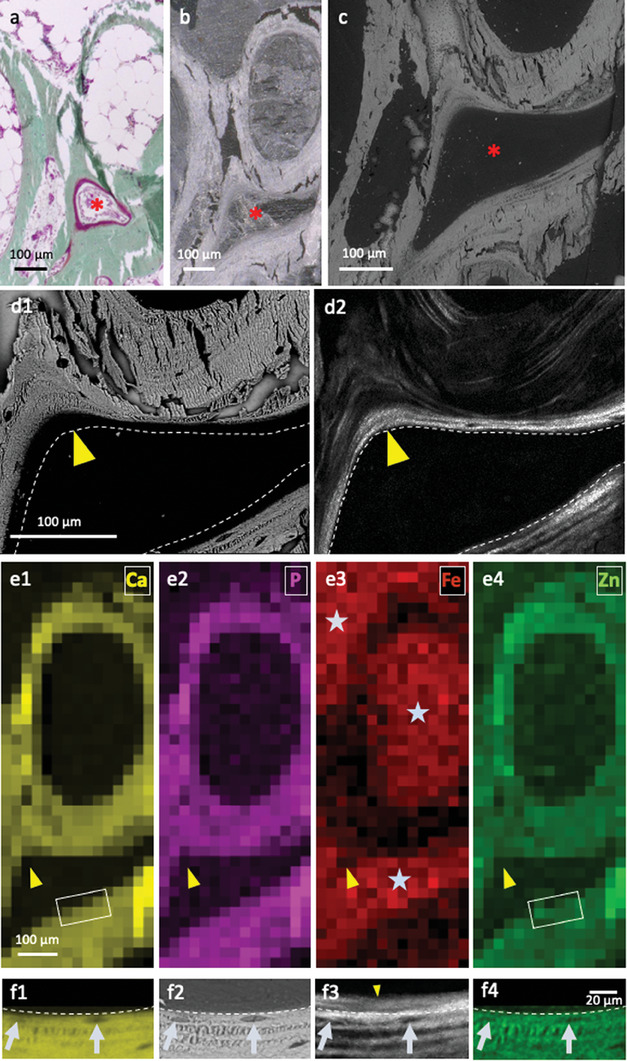
Identification of domains in interest on subsequent sections and different techniques, mineralized collagen and elemental mapping at moderate and high resolution. Observations by light microscopy of a histological thin section stained with Goldner trichrome a); the unstained subsequent thin section observed by PLM b) and backscatter SEM c). The red star (^*^) shows the same area. d1) Backscatter SEM and d2) SHG maps of the same region in a histological thin section of bone reveals the spatial relationship between lamellae and aOs leading to new bone formation (yellow arrowheads). Dashed line in panels b and a placed as a guide to the eye for direct comparison and interpretation of the different imaging contrasts. e) XRF spectra, matching panels including areas (d1) and (d2) (yellow arrowheads), provide complementary contrasts of the distributions of calcium (Ca), phosphorus (P), iron (Fe), and zinc (Zn). Note that Fe is found exclusively in bone marrow (grey stars) whereas Zn, despite its low density, closely matches the distributions of apatite mineral (Ca, P). f) Comparative imaging of the inset marked in e1,e4) by EDX f1, f4), backscattered SEM f2) and SHG f3). The chemical mapping of Ca (f1) matches the backscattered signal (f2) and the signal from Zn (f4) with osteocytes visibly embedded in the mineralized lamellae. The same cells are visible in SHG (f3) highlighting the existence of a ≈8 µm thick non‐mineralized and non‐fibrillar collagen layer of aOs juxtaposed on the bone.

Transition zones are relatively straightforward to image locally, but difficult to colocalize with complementary contrasts needed to compare findings over large tissue segments. Moreover, the use of subsequent sections for complementary analysis methods precludes straightforward correlation, as illustrated in **Figure** **5**a (light microscopy), b (unstained section PLM), and c (SEM) which shows that incremental (4 µm) sections exhibit small but significant structural differences (indicated by red ^*^ in multiple sections). We therefore integrate information obtained from backscatter SEM (Figure 5d1), SHG (Figure 5d2), and XRF (Figure 5e) of the same histological bone section. To correlate the non‐mineralized segments, we make use of the presence of small amounts of Fe and Zn in the tissue that help us align results obtained by the complementary imaging modalities. Despite some tears in the 4 µm‐thick slices, the high energy back‐scattered electron micrographs exhibit normal bony structures in the mineralized trabecula surrounding the bone marrow cavity (Figure 5d1). The same slice imaged by SHG (Figure 5d2) identifies the collagen of the aOs in relation to the MB. As a guide to the eye, a dotted white line and a yellow arrowhead demarcate the osteoid tissue which is not visible by the high‐energy electrons. The SHG signal arises directly from the collagen molecules in aOs as well as fibrils in the MB (Figure 5d2) and confirms that the organization of collagen (in its free molecular or packed fibrillar form) is continuous. Note that the SHG signal originates from the polarized collagen molecules and therefore it arises from both molecules and fibrils lying orthogonal to the incoming excitation laser, as shown also in vitro for lyotropic collagen phases.^[^ ^51^ ^]^ Curiously, collagen in aOs always has a brighter signal than SHG measurements of the adjacent mineralized tissue, possibly due to the mineral attenuating the signal emerging from the MB collagen.

In the exact same regions measured by backscattered SEM and SHG, 2D maps of high‐sensitivity XRF (Figure 5e) reveal perfectly matching distributions of Ca and P (panel e1‐2). Importantly, and unlike EDX, the XRF signal arises from the full 4 µm thickness of the bone slice, since spectra are stimulated by the incoming X‐ray beam and are not limited, as EDX is, to electron interactions on the outer 2 µm of the bone surface.^[^ ^52^ ^]^ XRF also reveals a signal of Fe (panel e3) arising from traces of blood in the bone marrow (grey stars), resulting in an inverse contrast as compared to mineralized bone (panel e1‐2). The outer margin of the bone marrow space is the point where new layers of collagen are identified by SHG (yellow arrowheads). Curiously, all regions with chemical signals of apatite are also accompanied by a signal of Zn that is higher than what the background levels in the organic tissue (panel e4). Indeed, XRF is extremely sensitive to the ppm range of elements such as Zn, known to exist in small quantities in mineralized bone tissue.^[^ ^53^, ^54^ ^]^ Panels f1 and f4 are high resolution EDX maps of the inset rectangular regions marked in e1 and e4. There is an excellent match between EDX and XRF despite the weak Zn signal. Importantly, the Ca chemical EDX map clearly reveals embedded cells (osteocytes) in the bone lamellae; this corresponds to the back‐scattered image in panel e2. Direct comparison of the same region observed by SHG (panel f3) highlights the presence of a non‐mineralized ≈8 µm thick collagen domain juxtaposed on the mineralized bone lamellae (compare d1 and d2, f1, f2, and f3, yellow arrowhead), well matching the LC Polscope results. These results demonstrate the significance of co‐aligning very different contrasts over large tissue segments to be able to describe the arrangement of the components of the tissue. These results confirm the 3D long‐range organization of the collagen outside of the MB. They also showcase the gradual transition from non‐mineralized to mineralized MB.

### 2.4 Proposed Mechanism for Bone Plywood Texturing

**Figure 6:**
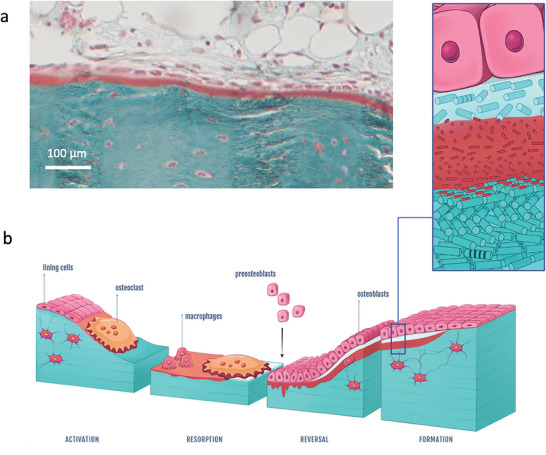
Alternative aOs/osteoblasts interface and schematic illustration of typical bone remodeling with the proposed mechanism for twisted plywood formation through acidic collagen mesophase. a) Light microscopy overview of a bone histological section stained with Goldner trichrome showing that nOs is not always seen; osteoblasts can be in contact with aOs. b) The schematic representation of the proposed mechanism for bone remodelling process includes the different steps observed here and matching observations described in the literature. (right) After the activation phase, bone resorption occurs via osteoclasts (multinucleate cell) through proteolytic enzymes and protons release (resorption phase). After further debris removal via macrophages, osteoblasts (mononuclear cuboid cells in pink) produce collagen fibrils (forming the newly osteoid tissue in light blue) (reverse phase) that progressively dissolve into or interact with the acidic ECM domains (in red) in which a collagen‐based mesophase is formed via molecule accretion. Thereafter, stabilization of the cholesteric geometry occurs through the co‐precipitation of collagen fibrils; MB is formed exhibiting a mineralized twisted plywood pattern (formation phase). (left) Schematic representation of the collagen molecules‐based domain (aOs) that co‐exists between two fibrillar tissues (nOs and MB).

Our results show previously‐overlooked evidence suggesting that twisted plywood containing bone comprises a state of matter that is an acidic collagen‐based mesophase gel (the aOs). Consequently, aOs is not always visible in osteoid production in other types of bone in different mammals where the osteoid thickness can vary significantly.^[^ ^7^ ^]^ The presence of a persistent acidic zone makes it possible for the cholesteric texture to mature and stabilize. One important example for when this acidity plays a role is documented, during the remodeling process (schematically outlined in **Figure** **6**b). We propose that there is an interplay between the biological activity of cells and the establishment of a low pH gradient that can be explained as follows:

Resorption of old bone material is induced by osteoclasts within local, defined acidic microenvironment^[^ ^55^ ^]^ (pH ≈3–4)^[^ ^34^ ^]^ (Figure 6b, activation phase). Different types of cells (including lining cells) participate in enzymatic digestion of old ECM components^[^ ^56^ ^]^ while macrophages clear away the debris (Figure 6b, resorption phase). Thereafter, pre‐osteoblasts appear (Figure 6b, reversal phase) triggered by osteoclast products.^[^ ^57^ ^]^ Recent findings show that osteoclasts remain on resorption sites in contact with osteoblast lineage cells,^[^ ^58^ ^]^ which is often observed in bone biopsies (Figure S2, Supporting Information). The pre‐osteoblasts mature into osteoblasts and form a cellular monolayer on the resorbed surface. They secrete nOs which is made of type I collagen formed through cleavage of the C‐ and N‐ terminal ends of pro‐collagen. However, previous work has demonstrated that the nOs is not seen in all osteoid layers^[^ ^7^ ^]^ as exemplified in Figure 6a. In bone regions that develop the twisted plywood motif, an aOs is formed where the acidity, likely preserved by the secretion of glycosaminoglycans and the remodeling canopy coverage,^[^ ^58^ ^]^ slows down fibrillogenesis thus promoting collagen fibril twist (Figure 6b, formation phase). As the concentration of collagen molecules increases, the gel reaches the transition threshold to the cholesteric LC (depicted in the enlarged schematic in Figure 6b). We propose that the formation of aOs and plywood geometries is a thermodynamically driven maturation process in the ECM that slows down fibrillogenesis to allow structuring of the tissue. This does not exclude local cellular mechanical tension on the tissue as proposed for in vitro^[^ ^15^, ^56^ ^]^ and in vivo^[^ ^18^ ^]^ models, nor do our observations contradict the emergence of other bone microgeometries such as disordered domains.^[^ ^41^, ^42^ ^]^ Citrate, and other NCPs^[^ ^3^ ^]^ are stable under acidic conditions and may play a so‐far poorly described physico‐chemical role in preserving the acidic state, which remains to be tested, for example, in mice lacking Slc13a5.^[^ ^32^ ^]^ Such molecules could maintain the local chemical gradient conditions that drive fibril precipitation.^[^ ^59^, ^60^ ^]^ The emergence of collagen fibrils might also be due to molecular concentrations exceeding 150 mg mL^−1[^ ^44^ ^]^ and is likely assisted by water retention within proteoglycans.^[^ ^61^ ^]^ At later stages of tissue formation and mineralization, bicarbonate from the blood^[^ ^62^ ^]^ might help neutralize the low pH zone at the transition into MB.

Though much remains unclear regarding the control, extent, and prevalence of aOs formation, we note the significance of coupling and thermodynamic advantage of establishing and maintaining a bilayer of neutral and acidic domains (nOs to aOs) of collagen in plywood‐texture newly formed tissue. The implications are substantial also for bone pathology, where disruption or absence of an intermediate molecular twisted‐plywood order could be clinically relevant for understanding various bone disease situations. This includes, for example, Paget's bone disease, where abundant osteoid shows an abnormal appearance and structure^[^ ^63^ ^]^ possibly as a consequence of an imbalance in aOs formation.^[^ ^6^ ^]^ Maintaining or restoring an intermediate acidic osteoid may therefore become a possible target for novel treatment modalities.

## 3 Conclusion

More than 50 years of histological investigations have provided hints to how collagen molecules create the twisted plywood texture of MB. No study to date has yet revealed how the different parts of the puzzle come together. The work presented here sheds light on how the architecture of fibrillar collagen is created providing good indications for the origin of long‐range twisted plywood pattern formation. Our observations in sheep bone biopsies suggest that texture emerges from thermodynamically driven molecular ordering of a liquid crystalline collagen phase, following the same principles (*i.e*., lyotropic properties) described for acidic assays in vitro. This observation could also hint at an additional potential role for the large amounts of citrate synthesized by osteoblasts, beyond its involvement with mineral formation that occurs somewhat later in the bone formation process.^[^ ^64^ ^]^ These findings complement observations found for DNA where such molecular ordering was demonstrated in condensed phases.^[^ ^65^ ^]^ Our work has implications for other liquid crystal‐like suprafibrillar architectures creating crystallites (chitin), whiskers (cellulose) or fibrils (collagen) in different biological structures (e.g., arthropod cuticles,^[^ ^24^, ^66^ ^]^ plant cell walls,^[^ ^67^ ^]^ cuttlefish^[^ ^68^ ^]^ and marine mussels^[^ ^69^ ^]^) since thermodynamically‐driven low‐pH liquid‐crystal ordering in mesophases may be a generic strategy for biomacromolecule re‐arrangement and structuring into highly‐organized twisted plywood solid structures.

## 4 Experimental Section

### Experimental Strategy

In accordance with protocols used for orthopedic research, a sheep model due to its similarities with humans with respect to weight, bone and joint structure, and bone regeneration was used.^[^ ^29^ ^]^ Because osteoid tissues were found in bone remodeling sites where newly deposited bone attaches to old bone surfaces,^[^ ^70^ ^]^ histological thin sections were collected (Figure S1a,b, Supporting Information) and analyzed (Figure S1c, Supporting Information) in and around the bone defects (e.g., Figures S1d,e and S2, Supporting Information). The degree of bone repair varies according to the depth of the defect and where it was extracted from (e.g., Figure S1d vs Figure S2a, Supporting Information). The osteoid domains, cut along different orientations with respect to the remodeling site, were occasionally larger than usually described (up to 20 vs 5 µm) (Figure S4, Supporting Information). Histological bone sections were obtained from undecalcified bone embedded in resin and stained using the complementary procedures described below. After identification of each domain of interest by stained microscopy, subsequent serial slices were either further stained (e.g., Figure 4a; Figure S3, Supporting Information) or they were analyzed with complementary optical/electron/x‐ray methods (e.g., Figure 4b and Figure 5a–c; Figure S8, Supporting Information).

### Bone Samples Preparation

All bone samples were harvested from 2‐year‐old healthy French ewes from negative control experiments taken from biomaterials biocompatibility tests. The work plan was reviewed and approved by the IMM Recherche Institutional Animal Care and Use Committee (IACUC) prior to the initiation of this study. The Animal Care and Use Committee of IMMR was registered at the CNREEA under the Ethics Committee n°37. The animal research center (IMM‐Recherche) received an agreement (n°75‐14‐01) by the “Direction Départementale de la Protection des Populations”. The studies were performed in compliance with the Principles of Laboratory Animal Care, formulated by the National Society for Medical Research, and the Guide for the Care and Use of Laboratory Animals, by the Institute of Laboratory Animal Resources (published by the National Academy Press, Washington, D.C, 1996), as amended by the Animal Welfare Act of 1970 (P.L 91–579) and the 1976 amendments to the Animal Welfare were followed. The surgical procedures were performed as described by Nuss et al.^[^ ^26^ ^]^ A total of eight‐hole defects (diameter = 8 mm, depth = 13 mm) were created with a drill into the distal and proximal metaphysis of the humerus and femur of each animal (2 ewes). After washing the bone cavity with saline solution, five defects out of 16 were left empty to heal: the wound was covered, and the skin was stapled. Eight weeks after surgery, the sheep were euthanized and both femoral and humeral bones were harvested, freed from all overlying tissues and the drill holes were identified. Following animals sacrifice, bone samples were cut perpendicular to the original drill hole using a band saw revealing the round original bone defect. Histological preparation was performed immediately after extraction (see Figure S1, Supporting Information).

### Bone Histological Preparation

Bone surrounding the surgical sites of the five empty defects undergoing natural‐healing was used for analysis. The samples were fixed in 4% paraformaldehyde solution, dehydrated with increasing ethanol baths and immersed for one week in a solution of butyl methacrylate, methyl benzoate, polyethylene glycol, and benzoyl peroxide resin. Polymerization was triggered by addition of N,N‐dimethyl‐toluidine and the samples were placed at −20 °C for 48 h to prevent any tissue degradation due to the exothermic reaction. In each embedded sample, 4 regions (2 outer/peripheral and two in the block center) were studied within series of 9–12 thin‐cut slices, as shown in Figure S1d (Supporting Information). To facilitate good matching between stained sections and adjacent unstained sections, four micron‐thick serial thin sections were cut using tungsten carbide knives. After placement on glass slides, The resin was removed using 2‐ethoxyethylacetate and sections for staining were rehydrated. This made it possible to use consecutive triplicate sections for matching analysis between histological staining and adjacent slices analyzed by SAXS, SEM, SHG, EDX, XRF, and TEM.

### Histochemical Staining

*a) Goldner trichrome (Haematoxylin, Fuchsine, Light Green)*: Sections (n = 60) were sequentially stained and washed in series: An initial haematoxylin Weigert solution stain was followed by staining with Ponceau fuchsine. Following treatment with a 1% phosphomolybdic acid solution, the sections were stained with a light green solution. The sections were then dehydrated in a graded series of ethanol and xylene solutions after which the slices were mounted on glass carrier slides with a resin mounting liquid. The basic haematoxylin was a useful histochemical marker of acidic osteoid, appearing red while fibrillar collagen was stained in blue/green (light green stain). In all stained regions in each slice, many red‐domains indicative of aOs were found.

*b) Hematoxylin‐Eosin*: Sections (n = 9) were immersed in haematoxylin solution, rinsed and stained with a 1% eosin solution. Washed sections were dehydrated with graded ethanol and xylene solutions followed by mounting. With such staining, nuclei appear purple, the cytoplasm pink and collagen appears pink–red.

c) *von Kossa‐ Van Gieson staining (VK)*: Sections (n = 9) were preincubated with 1% silver nitrate solution under UV light and washed with 5% sodium thiosulfate solution. The sections were counterstained with Van Gieson picrofuscine, washed and dehydrated with graded ethanol and xylene solutions followed by mounting. With this von Kossa staining, calcium deposits were highlighted in brown–black, osteoid appears pink–red and hematopoietic tissue appears in yellow.

d) *Basic Fuchsin (Ziehl's Fuchsin, Carl Roth, Germany)*: stock solution was diluted to 1/5 (v/v) in distilled water followed by immersion of section (n = 9). The basophilic stain highlights acidic histological moieties in magenta including mucopolysaccharides (glycosaminoglycans) and glycoproteins. It can also be used to track proteins in acidic pH systems and as a nuclear dye.

e) Double stain *Alcian Blue/Basic Fuchsin*: sections (n = 9) were immersed for 30 min in 1% (v/v) Alcian Blue 8GX at pH 0.5 (acetic acid).^[^ ^71^ ^]^ The stain was specific for acidic mucopolysaccharides (e.g., sulfated glycosaminoglycans) and at low pH appears violet.

f) *Universal indicator solution pH (*Honeywell Fluka, *pH 3–10)*: The sections (n = 9) were immersed for 10 min and rinsed with distilled water then dried at 70 °C for 30 min.

### Light Optical Microscopy

Observations were performed using a transmission Zeiss Axio Scope A1, equipped with crossed polarizers, a quartz first order retardation plate and a DXM 1200CCD camera and a microscope Zeiss Axio Imager M2 equipped with a differential interference contrast (DIC microscopy also known as Nomarski) and an AxioCam MRc digital camera (Figure 1h1–2 and Figure 5b; Figures S3 and S5c–e, Supporting Information). The images were processed using the ZEN software (Zeiss). Then, bone sections were examined by a polarizing microscope (Olympus IX71) equipped with LC‐PolScope using an objective (x40, N.A. = 0.6). The sample was probed by monochromatic elliptical lights of wavelength 546 nm at different settings of a liquid crystal retarder.^[^ ^39^ ^]^ At each pixel of the image (Figure 1i), LC‐PolScope calculates the azimuth of the director (see color wheel in Figure 1i) and the optical retardance (intensity) in the range (0–273) nm. An optical image of the measured section (Figure 1i2) helps identify the regions of interest (marked red, yellow, and green–purple) across collagen structures. The collagen molecular axis was orthogonal to the birefringence orientation.^[^ ^40^ ^]^ Therefore, orientation fields can be simulated from geometrical models of such molecular arrangements.^[^ ^24^ ^]^ The molecular orientation of a cholesteric phase was defined by its pitch $\vec{p}$, that can exhibit an angle θ with the cutting plane. The resulting birefringence azimuth and retardance graphs (Figure 1j1,2,3; Figure S1h, Supporting Information) were consequently the planar projections of cholesteric 3D structures. The in‐plane observed pitch was $p_{2D}=\left|\vec{p}\right|/\cos\theta$. Its planar wave vector was *q*  = 2π/*p*_2*D*_ . The projection of the birefringence has two orthogonal components: one perpendicular to the pitch Δ *n*_⊥_ =  Δ*n*.sin *qx* and one along the pitch Δ *n*_∥_ =  Δ*n*.sin θ.cos *qx*, with Δ*n* the phase birefringence. The retardance was ${\left(\Delta n_{⊥}^{2}+\Delta n_{∥}^{2}\right)}^{1/2}$ and the azimuth was tan ^−1^(Δ*n*_⊥_/Δ*n*_∥_). The observed birefringence can thus be simultaneously fitted by varying $|\vec{p}|$, θ, and Δ*n* (Figure 1j1,2; Figure S6h1, Supporting Information).

### Preparation of lyotropic Acidic Collagen Solution

Type I collagen molecules were extracted from rat tail tendons.^[^ ^72^ ^]^ The acidic collagen liquid‐crystal phase was prepared according to a procedure described earlier^[^ ^27^ ^]^ that combines injection and reverse dialysis processes but without inducing fibrils (i.e., fibrillogenesis). Briefly, a volume of a ≈3 mg mL^−1^ soluble acidic collagen solution (0.5 m acetic acid) was mixed with an apatite ion containing solution of 0.5 m acetic acid, 110 mm CaCl_2_, 33 mm NaH_2_PO_4_ and 33 mm NaHCO_3_. The pH was adjusted to 2.2 and the final concentration of the collagen solution was ≈1 mg mL^−1^ with an ionic strength of 165.9 mm. 30 mL of this mixture was continually injected in a closed dialysis chamber at 15 µL min^−1^ for 8 days. The bottom of the chamber contained a dialysis membrane with a molecular weight cut off of 12–14 kDa. The reverse dialysis process was set against polyethylene glycol (PEG, 35 kDa, Fluka) dissolved in 0.5 m acetic acid up to ≈300 mg mL^−1^. The flow of the collagen solution was controlled to maintain the same pressure on each side of the dialysis membrane. After injection of the total amount of collagen, the chamber was allowed to equilibrate for 4 days inside the PEG solution. This time was required to reach the final collagen concentration with increased viscosity.

### Estimation of Collagen Triple‐Helix Charges as a Function of pH

The charges were calculated by applying acid‐basic dissociation constant for all ionizable residues (Asp, Glu, His, Lys, Tyr, Arg):

**Table:** 

Amino‐Acid	charge at low pH	charge at high pH
Aspartic Acid	0	−148
Glutamic Acid	0	−236
Histidine	19	0
Tyrosine	0	−9
Lysine [20% hydroxylated]	69	0
Arginine	158	0

The apparent pKas of the aspartic/glutamic acid were sensitive to the local dielectric constant^[^ ^73^ ^]^ with variations that can reach four units in water.^[^ ^74^ ^]^ PKa shifts between +0.4 and +1.4 units were used in the simulation. The values do not change the analysis of the charge evolution significantly with a pH decrease from 7 to 5.5: The basic (cationic) residues, were not influenced by the pH, since the acid (anionic) residues can be protonated, causing a decrease of the negative charge. Noteworthy, this decrease was not sufficient to inverse the net charge of the collagen molecule which was still slightly negative at lower observed pH.

### Ultrastructural and Elemental Characterizations

For scanning electron microscopy (SEM), unstained bone histological thin sections were coated with 10 nm of carbon and imaged with an Hitachi S‐3400N SEM with an accelerating voltage of 10 kV. Energy‐dispersive X‐rays spectroscopy (EDX) was performed using an Oxford instrument X‐MAX detector (20 mm^2^). Other unstained bone samples were used for transmission electron microscopy (TEM), where the histological thin sections were removed from the slide and coverslip using xylene, for embedding in araldite. For comparison, the acidic collagen liquid‐crystal sample was prepared for TEM following glutaraldehyde fixation and post‐fixation with 2% osmium tetroxide for 1 h at 4 °C. These samples were then washed in a cacodylate/saccharose buffer solution (0.05 m/0.6 m, pH 7.4), dehydrated through successive ethanol baths and embedded in araldite. Ultrathin araldite sections (≈70 nm) obtained with an ULTRACUT 7 (Leica) were stained with uranyl acetate and observed using a FEI TECNAI G2 Spirit Twin electron microscope operating at 120 kV. Backscatter electron microscopy was obtained using a PhenomXL G2 (Thermo Fisher Scientific, Eindhoven The Netherlands) in a low vacuum (60 Pa) imaging mode with the four quadrant backscatter BSE, and an acceleration voltage of 15 kV. Complementary measurements were collected to facilitate alignment of the density and chemical maps, where Zn signatures were obtained by high‐energy (30 kV) mapping. This data was obtained in a high‐vacuum CamScan MaXim electron microscope collecting EDX spectra using a Bruker Xflash 6130 spectrometer, deconvoluted and processed by Esprit 2.0 (Bruker, Berlin, Germany).

### Scanning Small‐Angle X‐Ray Scattering

Small‐angle X‐ray scattering (SAXS) measurements were obtained at the mySpot beamline,^[^ ^75^ ^]^ synchrotron radiation facility BESSY II of the Helmholtz‐Zentrum Berlin für Materialien und Energie (HZB, Berlin, Germany). The domains of interest were identified with a metal ring attached to the unstained bone histological thin section, identified at high‐resolution using a light microscope. A 10 keV (λ = 1.2400 Å) X‐ray beam shaped using a 10 µm pinhole was used. The samples were mounted and displaced along y and z axes (lateral, vertical) performing line‐scans or 2D maps with step sizes of 10 or 20 µm. The circular illuminated area on the sample had a FWHM of ≈12 µm. The beam was monochromatized using the beamline Mo/BC DMM. The SAXS signal was acquired using an area sensitive CCD‐detector (MarMosaic 225, Mar USA), with 3072 × 3072 pixels where each pixel size was 73.242 µm^2^. The beam center and the sample‐detector distance were calibrated using the powder X‐ray diffraction pattern of silver behenate standard (s = 0.017 nm^−1^). Parasitic scattering from kapton films, pinholes and air was also collected for background correction. The measured patterns were transformed into radial or axial profiles as shown in Figure S8a–c (Supporting Information) for further processing. The SAXS signal was proportional, among other things, to the product of the contrast and the volume fraction irradiated by the X‐rays, due to the presence of mineral nanoparticles.^[^ ^46^ ^]^

### Synchrotron X‐Ray Fluorescence Mapping

Additional 2D maps of 4 µm thick sections were collected on the mySpot beamline using an XRF silicon drift detector (ASAS‐SDD KETEK, Germany) with a 100‐mm^2^ sensitive active area and 167.4 eV energy resolution was used to collect Ca spectra. Similar to EDX measurements but integrating over the beam size and full sample volume, peaks corresponding to the K lines of emission spectra of Ca, Fe, Zn, and P were used to produce maps of the elements with respect to the aOs.

### Second Harmonic Generation

Second Harmonic Generation (SHG) maps were recorded using a Leica SP5 II microscope (Leica Mikrosysteme Vertrieb GmbH, Wetzlar, Germany). A Spectra Physics Ti: sapphire laser (Mai Tai HP, Spectra Physics, Santa Clara, CA, U.S.A.) with 100 fs pulse width at 80 MHz and wavelength of 910 nm generated the flux needed to generate 2‐photon interactions. The SHG collagen signal was detected in the range of 450–460 nm. The excitation wavelength was optimized using rat tendon fascicles. Images were recorded using 40× and 63× water immersion objectives with numerical apertures of 1.1 and 1.2, respectively. SHG image stacks were recorded with 1 µm depth (*z*)‐steps followed by measurements of the autofluorescence signal (800 nm excitation, 450–570 nm detection range).

## Conflict of Interest

The authors declare no conflict of interest.

## Author Contributions

P.Z. and N.N. contributed equally to this work. M.R. and C. C prepared the bone histological sections and the synthetic samples; M.R. and C.D. prepared the histological staining; M.R., M.M.G.G., and N.N. performed the PLM observations and analyzed the bone histological images; B.H. prepared bone samples for electron transmission microscopy; M.R. and N.N. performed electron transmission microscopy; I.G. performed the EDX measurements; M.R., I.G., N.N. performed scanning electron microscopy; M.R., I.Z., and N.N. performed the SAXS measurements; M.R., N.B., I.Z., M.S, and N.N. analyzed the SAXS data; A.B., F.A. performed and analyzed the Polscope experiments; P.Z. performed the SHG experiments; P.Z. and I.Z. performed the XRF experiments; J.M.K., T.A., G.C., and N.N. obtained funding; M.R., F.A., P.Z., and N.N. wrote the paper; N.B., I.Z., P.F., T.A., M.M.G.G. critically reviewed the paper; P.F., P.Z., and N.N designed and revised the final manuscript; N.N. completed and coordinated the project.

## Supporting information

Supporting Information

## Data Availability

The data that support the findings of this study are available from the corresponding author upon reasonable request.
