# Supplementary material for: Acidic Osteoid Templates the Plywood Structure of Bone Tissue
Source: Adv Sci (Weinh). 2023 Dec 19;11(9):2304454. doi: 10.1002/advs.202304454 (PMC10916609; doi:10.1002/advs.202304454)
Supplement: Supplementary file 1 — Supporting Information [file ADVS-11-2304454-s001.pdf]

ADVANCED SCIENCE
Open Access

Supporting Information

for *Adv. Sci.*, DOI 10.1002/advs.202304454

Acidic Osteoid Templates the Plywood Structure of Bone Tissue

*Marc Robin, Chakib Djediat, Arnaud Bardouil, Niki Baccile, Camille Chareyron, Ivo Zizak, Peter Fratzl, Mohamed Selmane, Bernard Haye, Isabelle Genois, Jean-Marc Krafft, Guylène Costentin, Thierry Azaïs, Franck Artzner, Marie-Madeleine Giraud-Guille, Paul Zaslansky and Nadine Nassif**

Supporting Information

Acidic osteoid templates the plywood of bone material

Marc Robin, Chakib Djediat, Arnaud Bardouil, Niki Baccile, Camille Chareyron, Ivo Zizak[4], *Peter Fratzl, Mohamed Selmane, Bernard Haye, Isabelle Genois, Jean-Marc Krafft, Guylène Costentin, Thierry Azaïs, Franck Artzner, Marie-Madeleine Giraud-Guille, Paul Zaslansky*[§] *and Nadine Nassif*[§,*]

[§] Shared authorship

* To whom correspondence may be addressed. nadine.nassif@sorbonne-universite.fr

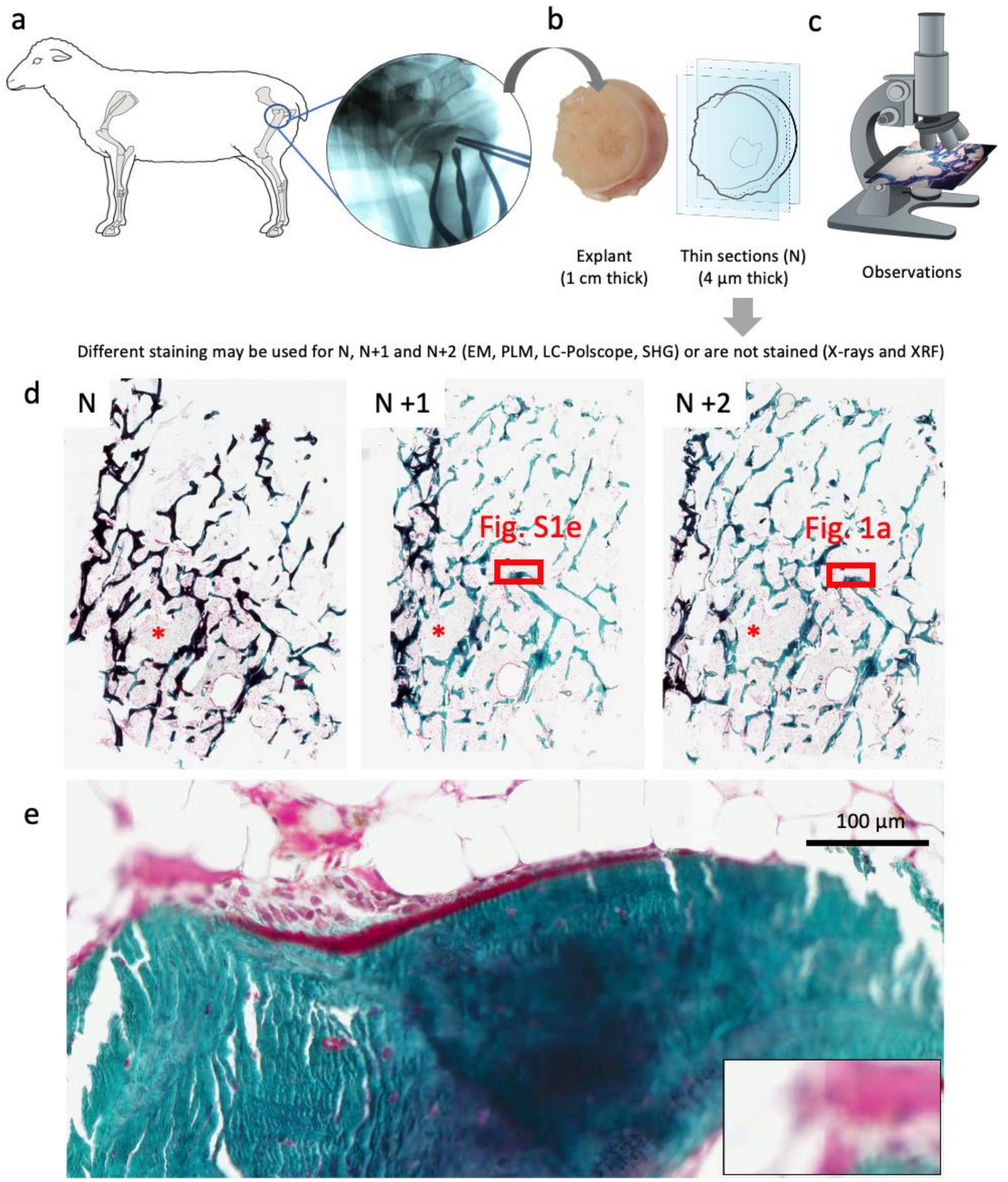

Fig. S1/ Experimental set-up. a, Schematic representation of the bone defect surgical procedure. Metaphyseal bone defects are performed on 2-year-old ewes using a drill (radiography, right). **b**, After 8 weeks, areas of interest are removed, cut into 4 µm serial thin sections (as illustrated schematically) and colored (or not) with different stains. **c**, Electron and polarized light microscopies, Polscope and SHG are performed on the resulting thin sections. Classical light microscopy on stained sections allows the identification of the domains of interest for synchrotron SAXS and XRF investigations. **d**, Light microscopy overview of successive histological thin section of bone tissue colored with Goldner's trichrome stain (noted

N, N+1 and N+2). The red star* indicates the defect. The size of the defect and the spongy bone network slightly vary along the depth of the explant. The red rectangles in N+1 and N+2 indicate the enlarged sections that are shown in (**e**) and **Fig. 1a**, respectively. Acidic osteoid tissue (aOs) is colored red due to basic haematoxylin dye of acidic domains. The stain strongly contrasts with collagen fibrils in mature bone seen in blue/green by light green stain. Note that automatic scanning was performed on the whole series of histological sections resulting in some artifactual blurred domains (the osteoclast in **Fig. 1a** cannot be observed in the lower right rectangle).

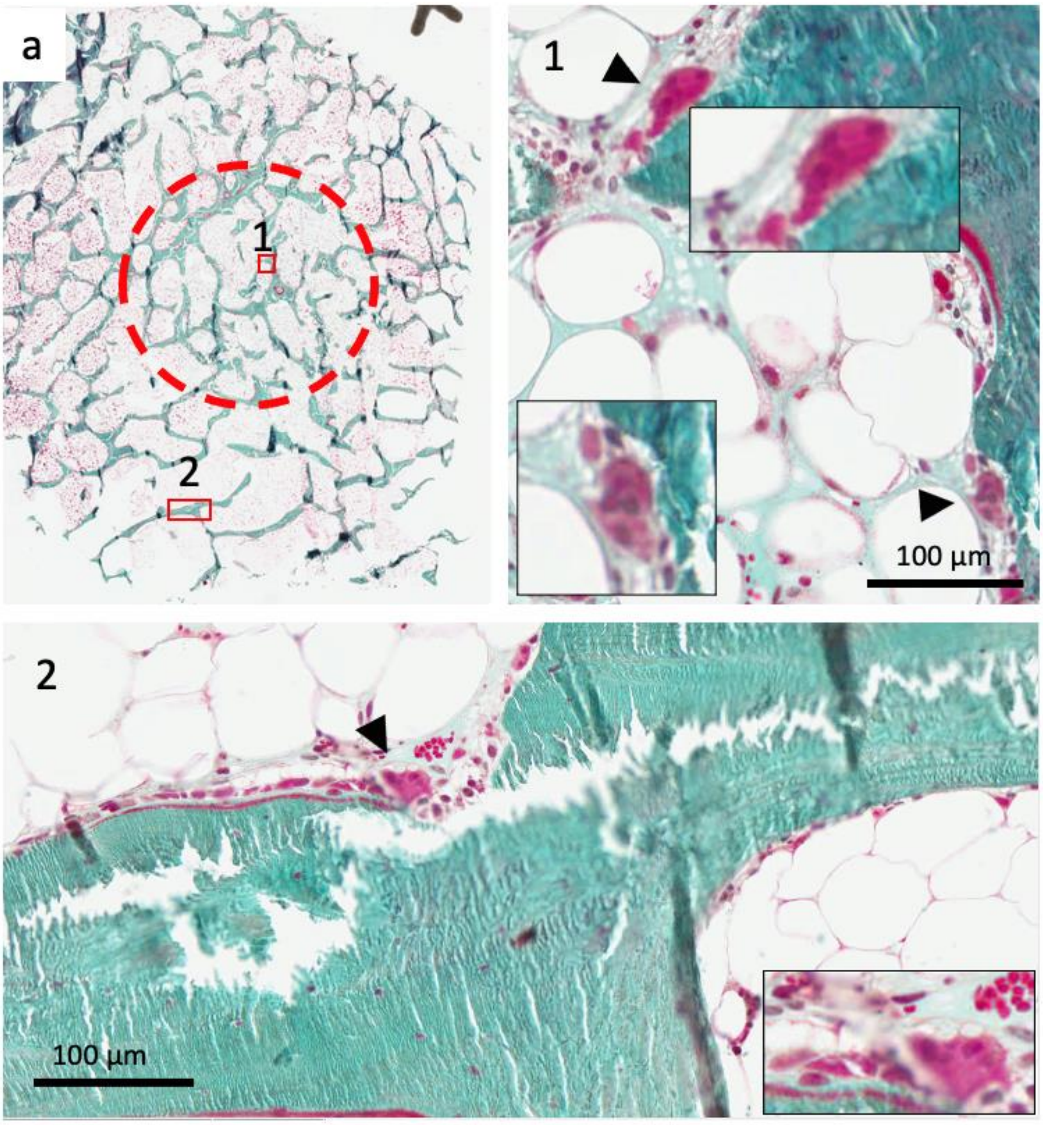

Fig. S2/ Identification of osteoclasts activity nearby remodeling site in and around the defect. Light microscopy overview (**a**) and local observation (**1-2**) of bone histological sections colored with Goldner's trichrome stain. The bone defect is fully repaired (dotted red circle). **1** and **2** images are higher magnifications of the selected regions in (**a**) by the red rectangles. In **1** and **2**, the black arrows show the presence of osteoclasts that are observed at higher magnification in the rectangles.

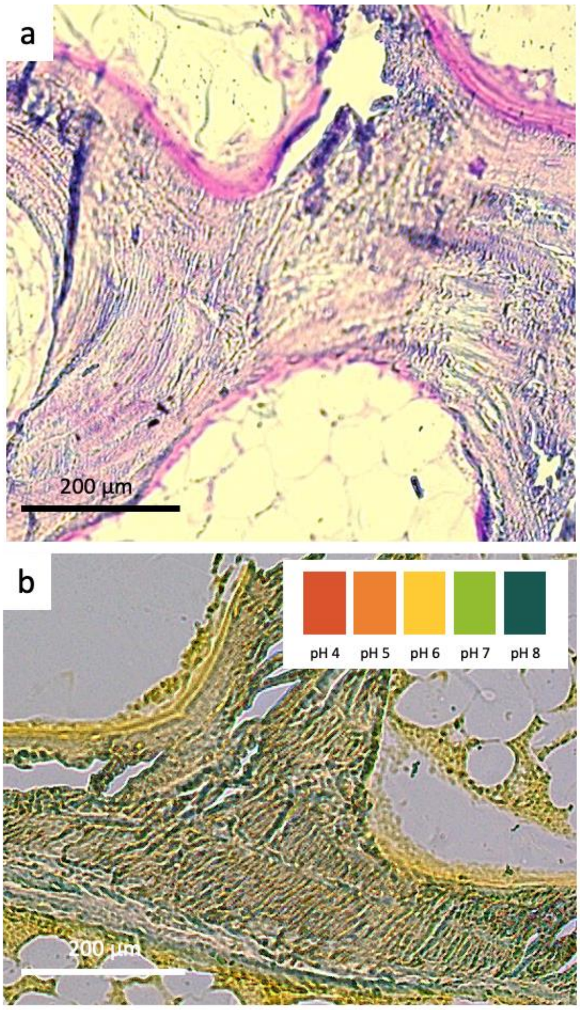

Fig. S3/ Investigation of aOs composition and pH. Histological thin sections of bone tissue colored with **a**, a basic fuchsin stain and **b**, a universal pH indicator essay (color chart in inset), observed with a DIC microscope.

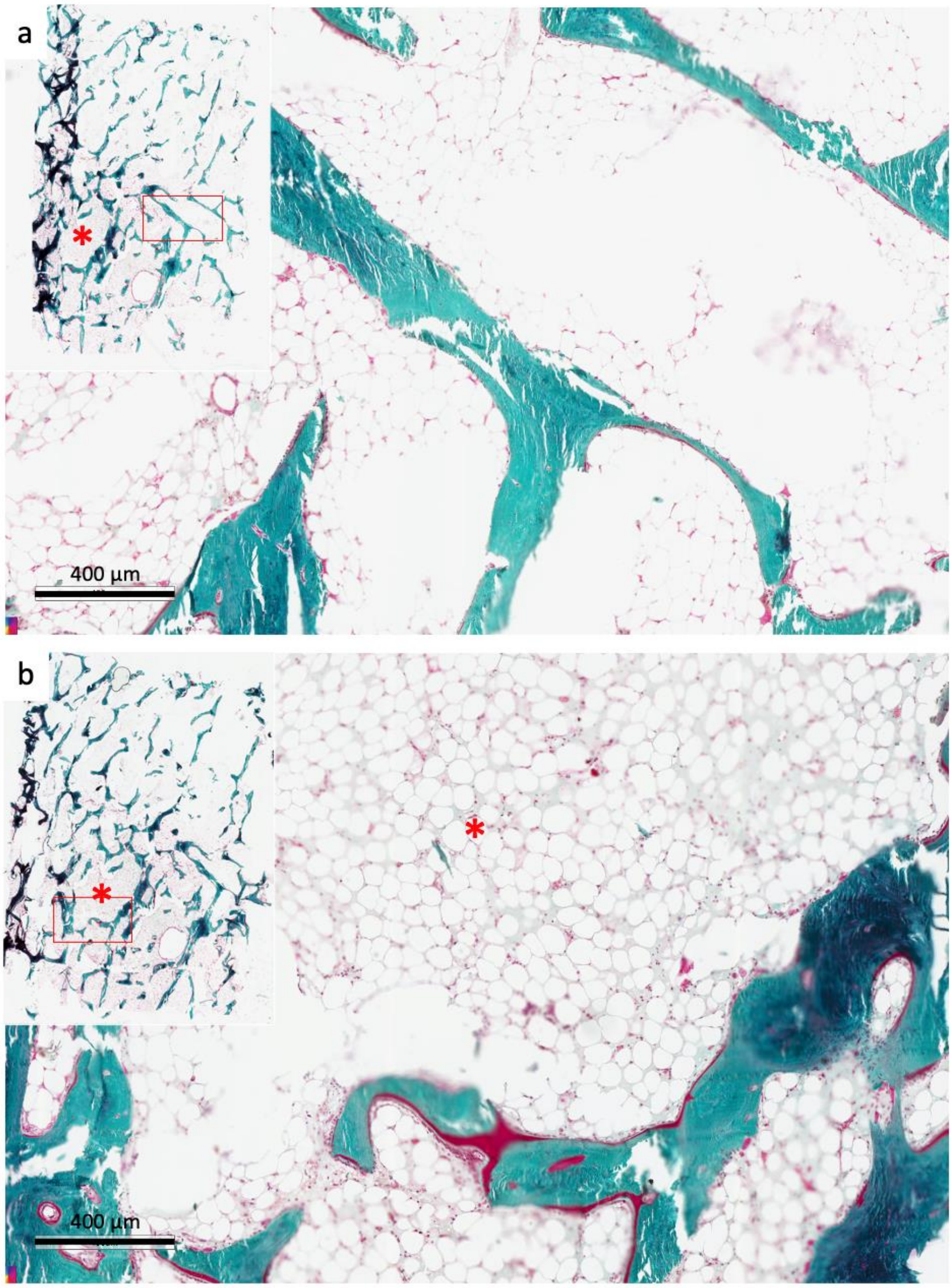

Fig. S4/ Investigation of the size of aOs size in relation to the location of the defect. Inset in **a** and **b**, Light microscopy overview of histological thin section of bone tissue colored with Goldner's trichrome stain. The red star* indicates the defect. The red rectangles delimit the areas that are shown at higher magnifications in (**a**) and (**b**). aOs red domains are larger in the vicinity of the defect (**b**) indicating an enhanced remodeling activity due to the recent chirurgical procedure.

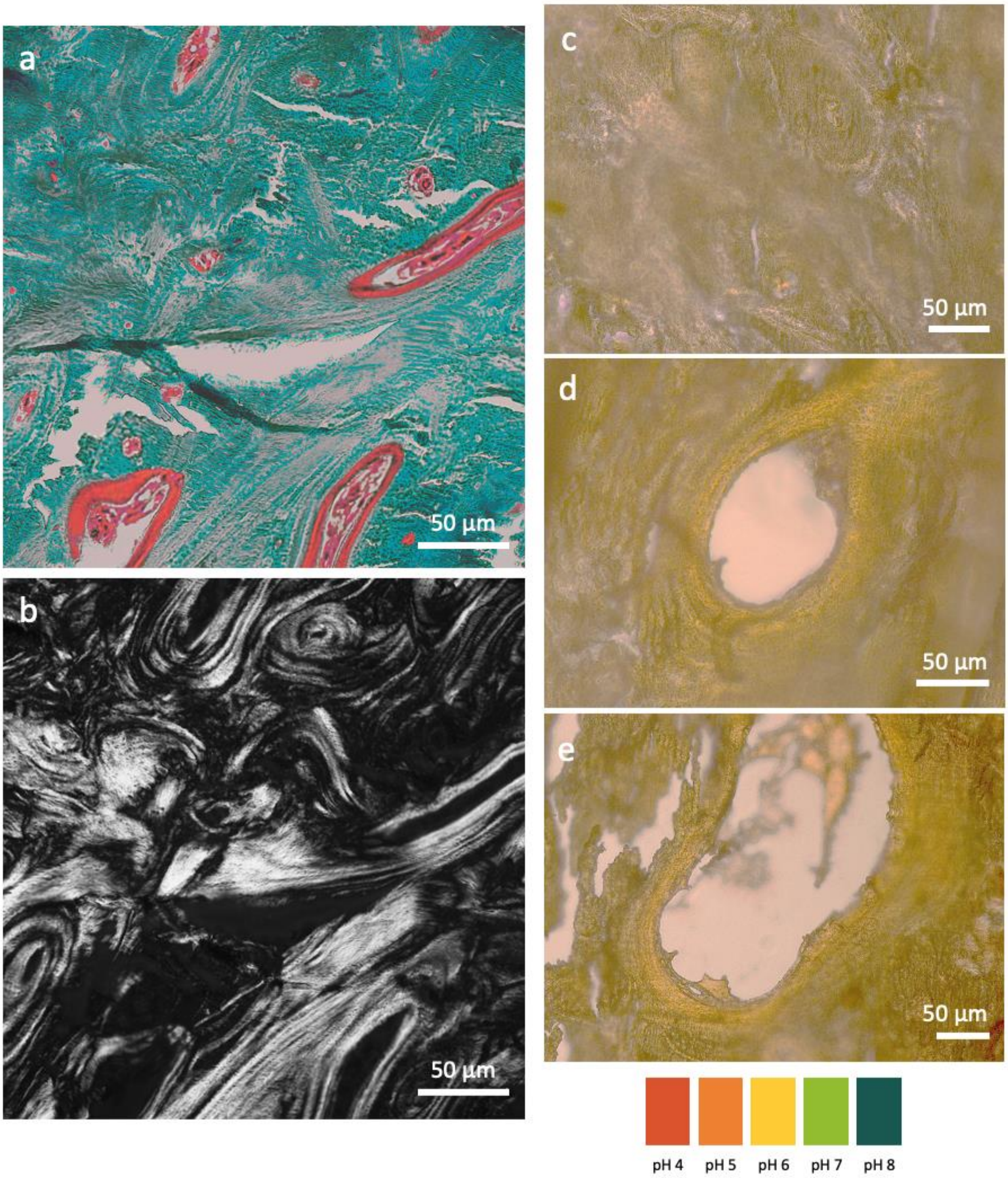

Fig. S5/ Histological thin section of compact bone tissue colored with Goldner's trichrome stain (a-b) and a universal pH indicator essay (c-e). a, Light microscopy overview of a compact bone histological thin section stained with Goldner's trichrome. Acidic osteoid tissue (aOs) is colored red due to basic hematoxylin dye of acidic domains. The stain strongly contrasts with collagen fibrils in mature bone seen in blue/green by light green stain. **b**, Same area as (a) observed under polarized light where due to birefringence, recurring domains of anisotropic collagen fibrils are seen with very different orientation all over the sample. **(c-e)** Light microscopy of a compact bone histological thin sections colored with a universal pH indicator essay (color chart below), observed with a DIC microscope at different magnifications.

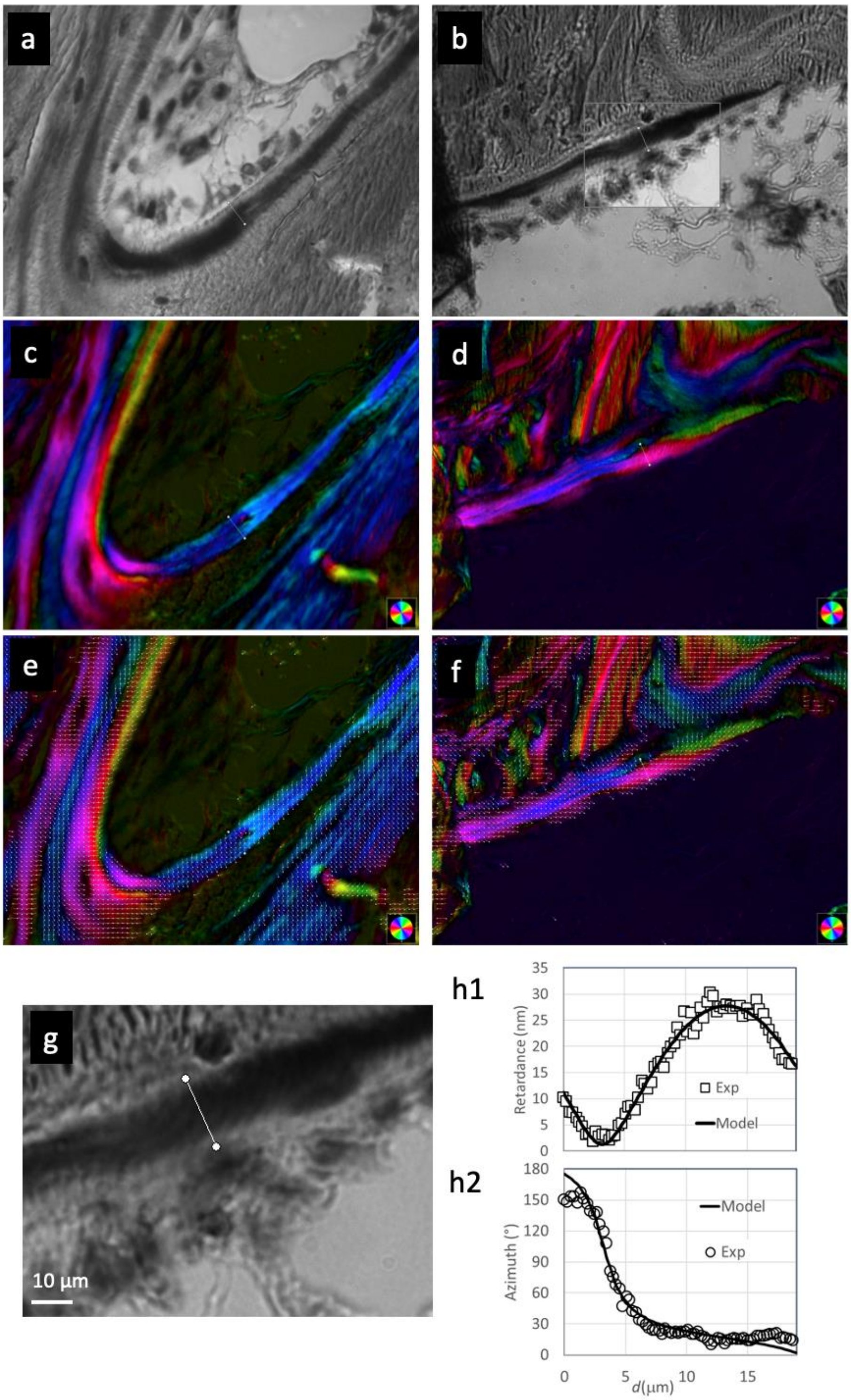

Fig. S6/ Depth analysis of the orientation and cholesteric pitch in example aOs regions. A distinct remodeling domain observed by light microscopy (**a,b**) and LC-polscope microscopy (**c,d**): the azimuth orientation is depicted by the color code and corresponding to the color wheel orientation. Optical retardance is provided by the Intensity. Birefringence vectors are overlayed in (**e,f**). The rectangle in **b** corresponds to the enlarged section shown in **g**. These are used to calculate **h1**, Retardance and **h2**, Azimuth of the birefringence measured along the continuous white line in the aOs; (**h1**, **h2**) fitted with a cholesteric model (see text) reveals an excellent match between simulation and measurement.

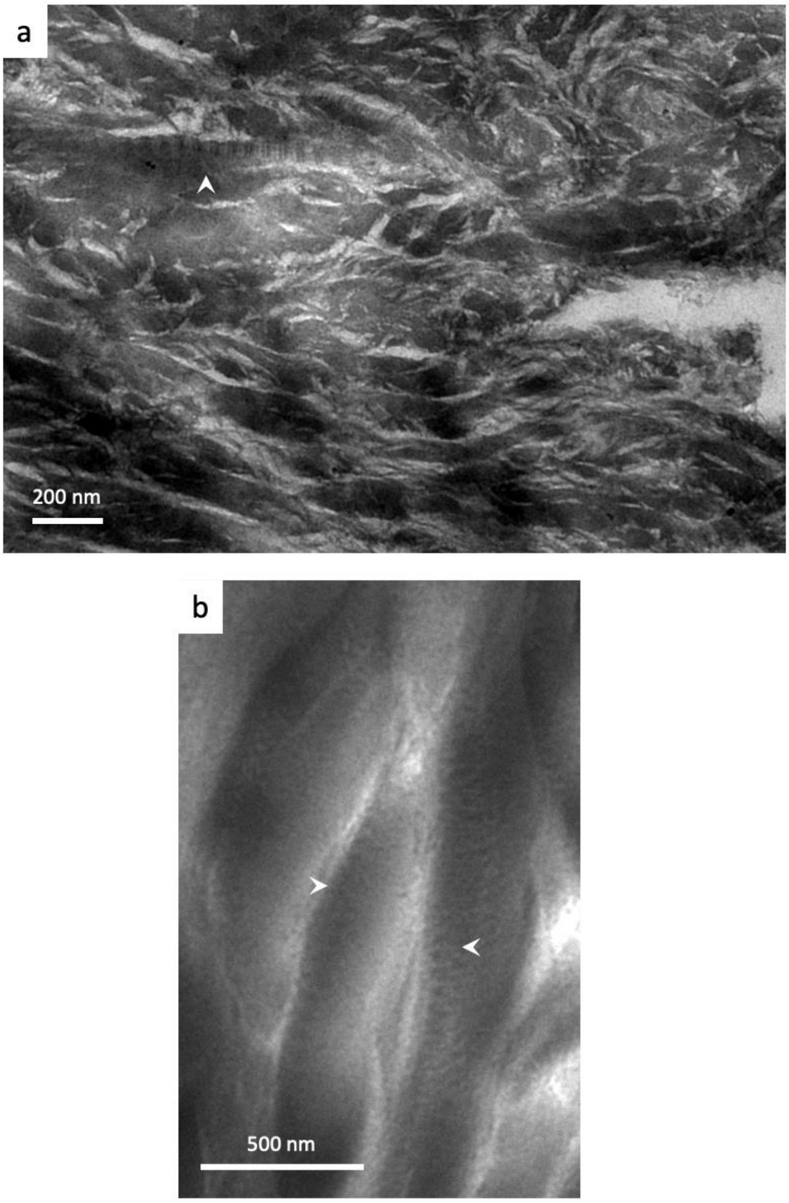

Fig. S7/ TEM investigation of collagen ultrastructure of aOs (a) and of *in vitro* acidic collagen mesophase containing apatite ion precursors. Both, aOs and the synthetic collagen mesophase appear dense to electrons with anisotropic (*e.g.* parallel) packing structures. Some fibrils are rarely observed (white arrows).

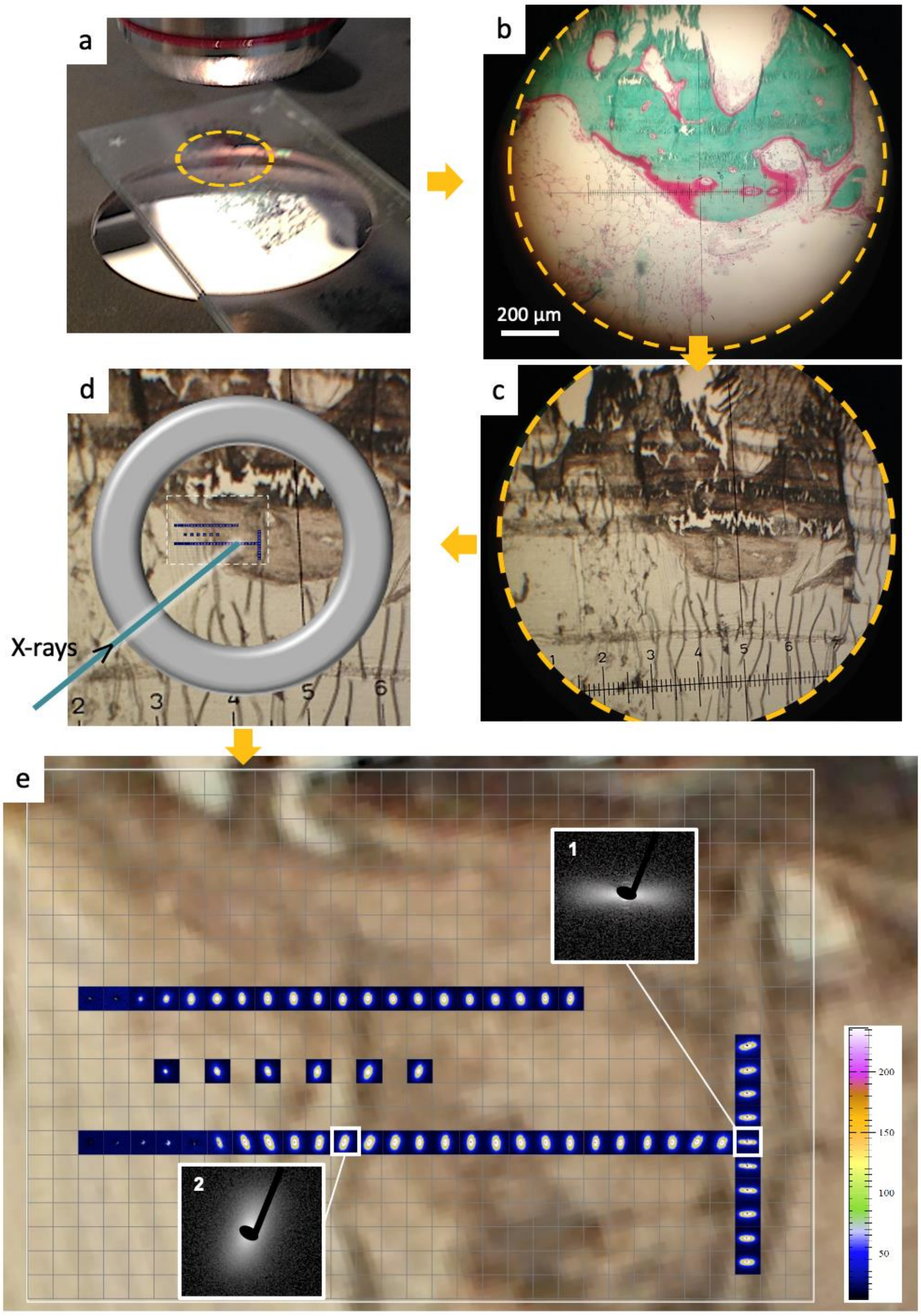

Fig. S8/ Selection of acidic osteoid domains under the microscope for small-angle X-ray scattering mapping and related spectra. a, Observation of the stained histological thin section under light microscope and identification of the domain of interest (dashed orange circle). **b**, Domain of interest seen through the ocular lens (N) **c**, Adjacent unstained thin section (N+2) used for SAXS measurements where the corresponding area of interest is observed. Unstained sections were mapped to avoid artefacts of staining. **d**, The area of interest is marked with a metal ring. **e**, 2D SAXS patterns corresponding to the signals orientations indicated in Figure 2b in the main text. The color coding indicates the intensity (arbitrary unit) of the radiation scattered. 2D SAXS pattern 1 corresponds to a signal recorded for mature bone. 2D SAXS pattern 2 corresponds to a signal recorded for the acidic osteoid domain. The scatter patterns are overlaid on an optical image of the unstained bone slice (**d**).
